# Mechanisms of aggregation in an ant-tended treehopper: Attraction to mutualists is balanced by conspecific competition

**DOI:** 10.1371/journal.pone.0181429

**Published:** 2017-07-21

**Authors:** Manuel A. Morales, Andrew G. Zink

**Affiliations:** 1 Department of Biology, Williams College, Williamstown, Massachusetts, United States of America; 2 Department of Biology, San Francisco State University, San Francisco, California, United States of America; Indian Institute of Science, INDIA

## Abstract

Understanding the spatial structure of populations and communities has been a dominant focus of ecological research, and spatial structure is increasingly seen as critical for understanding population dynamics. Habitat (or host) preference is a proximate mechanism that can generate aggregation or overdispersion, lending insight into the ultimate consequences of observed spatial distributions. *Publilia concava* is a univoltine phloem-feeding insect that forms mutualistic associations with ants, which consume honeydew and protect treehoppers from predation. Treehopper adults and nymphs are aggregated at the scale of goldenrod plant stems, and previous studies have suggested that this aggregation is an adaptive response that increases feeding performance or maximizes benefits of ant-tending. Previous studies have also shown experimentally that individual treehoppers preferentially oviposit on plants with ants present, but a complimentary hypothesis that treehoppers prefer to oviposit near conspecifics (e.g., to take advantage of density-dependent ant attraction) remains untested. We show that, as expected, the probability of treehopper oviposition increases with ant-presence and relative ant abundance. However, we also find that treehopper oviposition decreases with increasing treehopper density. Thus our results are inconsistent with the hypothesis that treehopper aggregation is a socially cooperative strategy to attract ants; we suggest that aggregation is a form of conflict and an unavoidable by-product of individual responses to ant-tending levels.

## Introduction

Understanding the distribution and abundance of organisms in space is at the core of ecological research [1], and spatial structure is increasingly seen as critical for understanding the population and evolutionary dynamics of species and communities [2]. For example, the conditions for coexistence in species interactions, including predation and obligate mutualism, depend critically on the degree and pattern of spatial structure [3–7].

Spatial structure can emerge *de novo* from species interactions or as a consequence of underlying abiotic heterogeneity, but such structure is nevertheless affected by the movement behavior of individuals. Despite this, the behavioral movement rules that drive spatial structure are still relatively understudied (reviewed in [8]). Importantly, understanding the proximate causes of movement behavior can often shed light on the ultimate evolutionary consequences of dispersal and habitat selection, improving our understanding of the effects of spatial structure for these populations (reviewed in [8]).

Host-visitor mutualisms represent a category of consumer-resource interaction in which visitors (i.e., consumers) provide some by-product benefit to a resource-providing host [9]. Host-visitor mutualisms include many familiar examples of mutualism, such as pollination, seed dispersal, and ant-protection interactions. For consumer-resource interactions in general, the response of consumers to spatial variation in resource density is a central component of ecological theory [10]. Examples include the resource concentration hypothesis [11], optimal foraging theory [12], and aggregation or functional responses [10]. For the case of host-visitor mutualisms, the functional and aggregation responses of consumers are influenced by density-dependent variation in the efficiency [13] or abundance of mutualist partners [14], respectively. While the aggregation response is distinct from the functional response, in that it is an implicitly spatial process, they are similar in that they both represent short-term behavioral responses by consumers to resource density [15].

One of the strongest conclusions to emerge from mutualism theory is that the benefit of mutualism should decline with higher densities [13,16]. As with consumer-resource interactions in general [10], patterns of host benefit will depend on the combined aggregation and functional response of the visitors [17–25]. Thus, for many mutualisms, spatial heterogeneity in mutualist abundance is fundamentally linked to patterns of density-dependent benefit and ultimately influences population dynamics.

The interaction between *Publilia* spp. treehoppers and ants has become a model system for studies of host-visitor mutualism. *Publilia* treehoppers benefit from ant tending both indirectly via protection from predators [25–27] and directly, probably via increased feeding rates [25,27]. Previous studies have shown that *Publilia* treehoppers receive maximum benefit at both low and intermediate densities of treehoppers [25–27] as mediated by competition for ants and patterns of density-dependent predation [14,25,28].

Despite theoretical predictions that benefit should decline at high densities and empirical observations showing maximum benefit to ant-protected herbivores at low densities [21,25,27], *Publilia* treehoppers are aggregated at the scale of host plants [29,30]. Two mutually non-exclusive hypotheses have been put forward to explain this distribution: First, treehoppers congregate with respect to conspecifics because treehoppers receive maximum benefit from ant tending at relatively high (e.g. intermediate) conspecific densities, with possible additional advantages of host plant overcompensation [26,29,31]. Second, the spatial distribution of treehoppers is generated by ant-dependent oviposition behavior of individuals attracted to ants alone, with conspecific aggregation emerging as a by-product of selection for ant-tending [28,32]. Ant-dependent oviposition has already been demonstrated experimentally for *P*. *concava* treehoppers [32], however the hypothesis that conspecific attraction also contributes to a clumped distribution of treehoppers was not tested in that study. Below we simultaneously evaluate the relative importance of ant-dependent oviposition versus conspecific attraction in driving aggregation of adult (and subsequently nymphal) treehoppers at the scale of host plants using individual mark-recapture methods [33]. Ultimately, these proximate mechanisms controlling the spatial distribution of treehopper females on host plants generates the variation in treehopper nymph abundance that drives patterns of density-dependent benefit, and ultimately the population dynamics of this mutualism.

## Materials and methods

### Natural history

*Publilia concava* is a univoltine phloem-feeding insect found primarily on tall goldenrod (*Solidago altissima*) for populations located in the northeastern United States. Adult females lay eggs in the spring, nymphs hatch out in early summer, and both adults and nymphs are tended by ants. Females guard eggs for up to three weeks and turn over offspring care to ants which begin tending the nymphs after they hatch [34,35]. Females initiate one or two broods within a variable time period but usually not exceeding one month between broods. Two broods from a given female are often on the same host plant and multiple females often oviposit on a single host-plant. Nymphs and newly eclosed adults remain on their natal host plant so that aggregations from a few to over 1000 nymphs (resulting from broods of one to over ten females) persist throughout the period of summer development. Thus, the population density of treehopper nymphs and their density-dependent survivorship depends on the initial oviposition behavior of females. Additional life-history details of *P*. *concava* can be found in [27,35,36].

### Monitoring naturally occurring aggregations

Data used in these analyses come from three sites—two located in north central New York and one located in northwestern Massachusetts, USA. The data from New York sites focused on two adjacent populations of *P*. *concava* on either end of an abandoned agricultural field near Newfield, NY, with permission to use the site granted by R. Bergman. One population was clustered around a mound of the ant *Formica exsectoides* and the other around a mound of the ant *Formica subsericea*. Adult males and females emerged in both NY populations in mid-May of 1999. By the end of May males had mostly disappeared and females could be found clustered on the plant stems. During this brief window of time between mating and the initiation of egg-laying (May 28–29, 1999) all plants containing *P*. *concava* adults, in both populations, were marked with flagging.

On the evenings of these same two days all adults were collected and all individual females within each plant were each placed in a solitary separate vial. Vials were refrigerated and each female was marked on the pronotum with a unique color combination using UniPaint Fine Line paint markers. By using up to ten colors and various orientations of four dots per female we were able to distinguish among all individuals studied in the field (N = 231 at the “*exsectoides”* site, N = 285 at the “*subsericea”* site, N = 516 total). Females were returned to their exact original plants (from which they were taken) during the very same evening that they were collected (usually within three hours). On the day after marking (May 29 and May 30 respectively) a small subset of females were found on adjacent plants; these females were returned to their original plant in an attempt to compensate for any disturbance caused by the previous night's marking.

From June 1 through June 20 the location of each female was recorded daily including the plant ID and her presence on individually tagged egg masses. Marked plants were searched daily for any new egg masses. Eggs are laid in a discrete clutch on the underside of a leaf, and usually only one female is associated with newly discovered eggs [37] although subsequent females may occasionally add eggs to the mass after the first day [35]. For the purposes of this study we attributed an oviposition decision to the female that was present on the first day an egg mass appeared (i.e. the initiator of the egg mass). Throughout the experiment any unmarked females that appeared on the marked plants were noted and then removed and placed on a nearby plant, almost always before they had laid eggs themselves. A small fraction of the originally marked females moved to nearby, unmarked plants, and these plants were included in future monitoring of marked female location. However, for the entire 20 days of this study there were no females that initiated a second clutch, either on a new plant or on the original plant where they laid their first eggs.

For each day (June 1–20) we recorded ant numbers immediately upon arriving at a marked plant, but before recording female location and egg masses to minimize disturbance to ants. We also recorded the species of ant present on that plant for each day. Species of ant corresponded with that of the adjacent ant mound, with the exception of *Lasius niger* (“*exsectoides* site”). Ultimately, for each of the twenty days of this study, we collected data on the oviposition decisions of marked females, the ant species and number of ants associated with any given female treehopper's plant, and the total number of females on the plant.

In Massachusetts the study site was an abandoned agricultural field located in Hopkins Memorial Forest, Williamstown Massachusetts, USA (42° 43' 34'' N, 73° 13' 25'' W) with permission to use the site given by the Hopkins Memorial Forest Users Committee and Williams College. Details of the site can be found in Morales and Beal [36]. An experimental “arena” was established to facilitate the tracking of individually marked treehoppers while minimizing disturbance. Parallel lanes (0.7m wide) spaced 1.7m apart on center were mowed within a 15m × 15m area. This allowed for observation of treehoppers without disturbing females on existing host plants.

Treehoppers were collected from a nearby site (32 each of males and females) and individually marked as described above. Treehoppers were released in four central locations within the experimental plot on 6 June 2005. Censuses of treehopper location were taken twice daily until 10 June after which censuses were taken daily until 20 June. At this point the majority of treehoppers had oviposited on a plant, left the area of the site, or otherwise remained unfound. Additional censuses were taken approximately every other day until 6 July. By the end of the census period at least one observation of location was made for 30 of the females and 23 of the males. At each census the location of the plant was recorded along with the presence of other ants and the number of other females on that plant. Additional data that were recorded included whether a given treehopper was observed mating, ovipositing, or guarding eggs, and the percent nitrogen of a leaf collected at the end of the observation period from each visited plant (taken from an unoccupied leaf approximately one-third of the distance down from the top of each plant, and measured using a Costech elemental analyzer).

### Analyses

All analyses were performed in the statistical environment R [38]. In particular, the probability of oviposition by treehoppers was estimated from marked individuals using mixed effects generalized linear models [39]. We used binomial errors to account for the binary response, and included plant and treehopper ID as random effects to account for the repeated-measures design. At the New York sites predictors of oviposition included day, site, the number of female treehoppers and ant abundance (the last two were calculated for each treehopper-plant combination over the period that a treehopper first arrived on a plant until it left that same plant or oviposited, whichever came first).

Because previous studies have shown that treehoppers benefit from ant tending in proportion to both the total number of ants per plant and the per-capita ant-tending level (which are correlated; [25,27], we used AIC to evaluate which of these predictors to use in our analysis of oviposition. The model including per-capita ant-tending levels substantially improved model fit relative to a model that included total ants per plant (ΔAIC = -3.4) and results presented below use this measure of ant abundance. Importantly, the per-capita ant-tending level is also much more likely to be the environmental variable most easily assessed by treehopper females that may not be able to assess overall ant levels at the scale of an entire plant.

At the Massachusetts site, predictors of oviposition included day, percent leaf nitrogen, whether the female was “guarded” by a male, and treehopper or ant presence on the plant. Because both treehopper and ant densities were low at the Massachusetts site, presence or absence rather than total number was used in the analyses.

## Results and discussion

**Fig 1:**
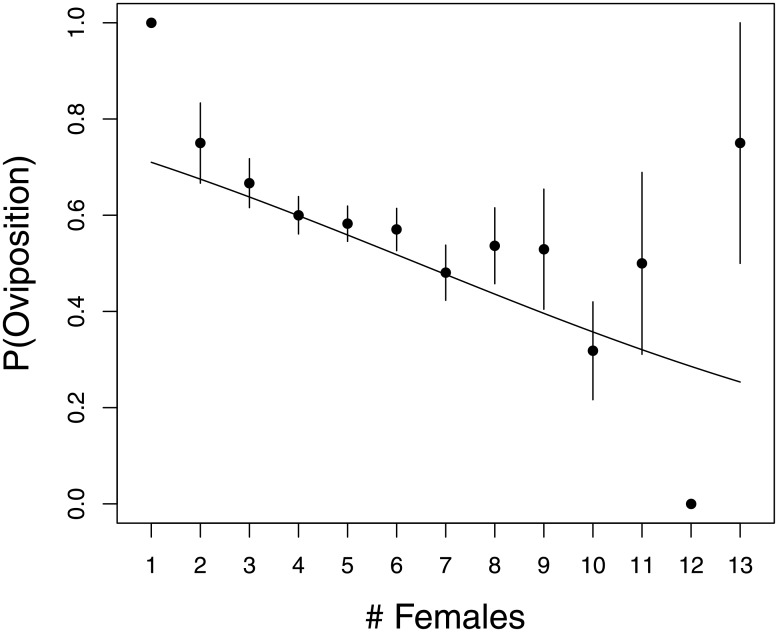
Probability of oviposition as a function of the total number of females per plant for the NY sites.

At both the Massachusetts and New York sites there was a strong positive effect of ant presence or relative abundance, respectively, on the probability of oviposition (Tables 1 and 2). In contrast, oviposition was a declining function of treehopper density at the New York site (Table 1, Fig 1), and was not influenced by co-occurring treehoppers at the Massachusetts site (Table 2). Similarly, neither leaf nitrogen levels nor mating status significantly influenced the probability of oviposition, for plants on which treehoppers were observed, at the Massachusetts site (Table 2).

**Table 1:** Parameter estimates and significance estimates at the two NY sites for the probability of oviposition by female treehoppers as a function of per-capita ant-tending level and treehopper density on a plant.

Parameter	Estimate^a^*	z-value	p-value
Intercept	0.98 ± 0.38	2.569	0.01
Δ Site (subsericea)	0.3 ± 0.22	1.403	0.161
Day	-0.0.7 ± 0.02	-3.318	<0.001
# Females	-0.16 ± 0.05	-3.154	0.002
# Ants / Female	0.63 ± 0.26	2.415	0.016

^a^Log-odds

**Table 2:** Parameter estimates and significance estimates for the probability of oviposition by female treehoppers at the MA site.

Parameter	Estimate^a^*	z-value	p-value
Intercept	-11.8 ± 4.7	-2.513	0.012
Δ Mate Guarding	2.37 ± 1.76	1.347	0.178
Day	0.64 ± 0.23	2.73	0.006
% Nitrogen	2.81 ± 2.04	1.377	0.169
Δ Females (presence)	-0.19 ± 1.2	-0.161	0.872
Δ Ants (presence)	3.18 ± 1.26	2.529	0.011

^a^Log-odds

There was a strong phenological signal at both sites as measured by a significant day effect (Tables 1 and 2) although the direction of this effect was reversed across sites—no females oviposited in the first two days of the study at the Massachusetts site while no females oviposited during the last three days of the study at the New York sites. Thus, our results likely span a discrete and defined period of egg laying in this species.

To evaluate the effect of ant species on the probability of oviposition, the analysis was repeated with the New York data set for the “*excsectoides”* site alone—the only New York site where multiple ant species were recorded. There was no significant effect of ant species on the probability of oviposiiton (Species + Species × #Ants / Female | Day + #Females + #Ants/Female: χ^2^_DF = 2_ = 1.842, *P =* 0.3981). Otherwise, results qualitatively matched that of the full analysis (Table 3).

**Table 3:** Parameter estimates and significance estimates for the probability of oviposition by female treehoppers as a function of ant species at the NY *exsectoides* site.

Parameter	Estimate^a^*	z-value	p-value
Intercept	0.32 ± 0.57	0.556	0.578
Day	-0.07 ± 0.03	-2.112	0.035
# Females	-0.16 ± 0.08	-1.996	0.046
# Ants / Female	1.55 ± 0.54	2.891	0.004
Δ Ant species (*Lasius niger*)	0.41 ± 1.79	0.228	0.819
Ant species × # Ants / Female	-0.83 ± 1.05	-0.793	0.428

^a^Log-odds

Because individual females sample host plants and choose whether to emigrate or oviposit based on local conditions, our analyses are analogous to those in which individual movement decisions are correlated with local environmental conditions [33]. Notably, previous studies have shown experimentally that treehoppers use ant presence as a cue for oviposition [32]. Because ants and treehoppers respond behaviorally to changes in densities of each other (e.g. removing ants will cause treehoppers to leave making any measure of conspecific effects irrelevant), we were not able to simultaneously manipulate ant and treehopper densities. Rather, we build on previous experimental work in this system to evaluate the movement and oviposition behavior of individual treehoppers under natural conditions.

Our results add to previous studies showing that treehoppers, like other ant-tended insects, use ants as an ovipositional cue [32,40,41]. In particular, we show that ant-tended treehoppers are more likely to oviposit than are untended ones (Massachusetts site), and that the probability of oviposition increases with the number of ants per female (New York sites).

In contrast, the probability of oviposition was unaffected by female presence at the Massachusetts site and was a declining function of female density at the New York sites. Thus, our results are inconsistent with the hypothesis that conspecific attraction during oviposition (i.e. congregation) drives aggregation in this system. At the Massachusetts site female densities were low—more than one female was noted on only 10 percent of plants and less than two percent of plants had more than two females. This difference in treehopper density may explain the above difference in effect of female density between the Massachusetts and New York sites. Interestingly, in NY populations the subset of suitable host plants is low because of heterogeneity in plant quality; this (like ant-tending) could increase female aggregation and neutralize conspecific avoidance. Another mutually non-exclusive possibility is that the pattern of female aggregation is a byproduct of individual selection for ant tending. Here, aggregation emerges because once a treehopper-ant pair discover each other on a given plant, both partners are more likely to stay, thereby increasing the probability that a subsequent treehopper arriving on that plant will encounter ants (despite there being a competing female nearby). In both of the above hypotheses, aggregation is actively avoided but nevertheless emerges as an inevitable consequence of strong selection for increasing ant-tending levels.

Previous authors have suggested that ant-tended insects may aggregate into groups to cooperatively optimize ant-tending levels [31,42,43]. We suggest that aggregation strategies may differ fundamentally between ant-tended insects that pay a significant cost for ant protection [44,45] and those that don’t [36,45,46]. In particular, ant-tended Lepidoptera (who pay a cost for protection via secretions) may mediate the cost of ant investment by aggregating because per-capita tending levels (and thus frequency of secretions) decline at high density [47]. In contrast, Hemiptera that benefit from ant-tending in part through increased feeding efficiency and where there appears to be little cost of ant-tending (e.g. *P*. *concava*; [27,36]) may be most successful at low densities where per-capita tending levels are high. This hypothesis makes the specific prediction that ant-tended insects that pay a significant cost for ant-tending (e.g. Lepidoptera) may show congregative behavior during oviposition, in contrast to the results presented here for *P*. *concava*, where females avoided aggregation.

Our findings suggest that, after controlling for per-capita ant-tending levels, females are less likely to oviposit on a plant when the density of treehopper females increases. This raises the question of how females can assess the presence of conspecifics; possible mechanisms include interactions with brood parasites [37] or vibrational signals [48,49]. We also found that treehoppers respond more strongly, in terms of oviposition behavior, to per-capita (female) ant tending levels rather than total ant levels on a plant. This may be because treehoppers assess ant presence directly through their own total contact time with ants (which would be proportional to the number of ants per treehopper rather than the total number of ants on a plant (*per se*). Importantly, any oviposition response to ant tending rate may not necessarily correlate perfectly with higher per-capita ant number (e.g. if instead higher interaction rate represents greater tending effort by individual ants). Alternatively, the response of treehopper oviposition to per-capita ant levels may be because treehoppers benefit in direct proportion to the per-capita number of ants present simultaneously [25]. This would suggest that ant tending provides direct benefits at the scale of single treehoppers [25,27], and that tending by two versus one ant increases female fitness (either directly, or indirectly through increased egg survival [35]).

Following nymph emergence, treehopper density per plant is positively correlated with both the overall constancy of ant-tending and the total number of ants on a plant over time [14,28], but it is associated with a decrease in per-capita tending levels [14,27]. Over the period of this study, however, the correlation between female density during oviposition and per-capita tending levels was low (*r* = 0.08 for the model analysis in Table 1) indicating that multicollinearity was not an issue in this analysies [50]. Thus, we were able to use multiple regression-based approaches to show that **both** low female density and higher per-capita tending rates increased oviposition. Ultimately, the aggregated pattern of female treehoppers per plant suggests that selection for maximizing per-capita tending levels or host-plant quality (i.e., selecting host-plants with few conspecifics) is at least partially offset by selection to maximize the likelihood of ant-tending (i.e. selecting a host-plant with ants despite the presence of conspecifics). These results suggest that female assessment of group size and subsequent competition may undermine, to some extent, the stability of the mutualism with ants (or at least the benefits that treehoppers receive). Theory suggests that mutualisms can be further stabilized when such costs (in terms of conflict within a partner species) are mitigated by inclusive fitness benefits due to kin cooperation [51, 52]; future work should investigate the possibility that female aggregation, given its potential costs at higher densities, is a form of kin cooperation in addition to an emergent outcome of ant attraction.
